# Clinical outcomes following surgical mitral valve plasty or replacement in patients with infectious endocarditis: A meta-analysis

**DOI:** 10.3389/fsurg.2022.1048036

**Published:** 2023-01-06

**Authors:** Song Wang, Ting Zhou, Jinhui Bian, Geng Li, Wenjing Zhang, Si Chen, Yefan Jiang

**Affiliations:** ^1^ Department of Cardiovascular Surgery, The Affiliated Taizhou People's Hospital of Nanjing Medical University, Taizhou School of Clinical Medicine, Nanjing Medical University, Nanjing, China; ^2^ Department of Cardiovascular Surgery, The First Affiliated Hospital of Nanjing Medical University, Nanjing, China; ^3^ Health Management Center, The Central Hospital of Wuhan, Tongji Medical College, Huazhong University of Science and Technology, Wuhan, China; ^4^ Department of Cardiovascular Surgery and Heart Transplantation, Union Hospital, Tongji Medical College, Huazhong University of Science and Technology, Wuhan, China; ^5^ Department of Ultrasound Medicine, The Second Afliated Hospital of Harbin Medical University, Harbin, China; ^6^ Department of Ultrasound Medicine, Union Hospital, Tongji Medical College, Huazhong University of Science and Technology, Wuhan, China

**Keywords:** infectious endocarditis, mitral valve plasty, mitral valve replacement, clinical outcomes, meta-analysis

## Abstract

**Background:**

For degenerative mitral disease, more and more evidences support that mitral valve plasty (MVP) has much better clincial outcomes than mitral valve replacement (MVR). However, the advantages of MVP in patients suffering from infectious endocarditis (IE) are unclear. To evaluate the appropriateness of MVP in IE patients, we conducted this meta-analysis. Based on the difference between active and healed phase, we not only compared the result of patients with IE, but also identified the subgroup with active IE.

**Methods:**

We systematically searched the clinical trials comparing clinical outcomes of MVP and MVR in patients suffering from IE. Relevant articles were searched from January 1, 2000 to March 18, 2021 in Pubmed and Cochrane Library. Studies were excluded if they were with Newcastle–Ottawa Scale (NOS) score less than 6 or lacking of direct comparisons between MVP and MVR.

**Results:**

23 studies were involved and 25,615 patients were included. Pooled analysis showed fewer adverse events and early or long-term death in the MVP group. However, more reoperations existed in this patient group. And the reinfection rate was close between two groups. Similar results were observed after identifying active IE subgroup, but there is no difference in the freedom from reoperation due to all-events.

**Conclusions:**

Although limitimations exited in this study, patients suffering from IE can benefit from both MVP and MVR. For surgeons with consummate skills, MVP can be the preferred choice for suitable IE patients.

## Introduction

Treatment for infective endocarditis consists of antibiotic therapy and surgery (1). Surgical intervention is needed in about half of patients suffering from IE (2). The indications for surgery in IE are well defined but the choice of surgical procedures is less defined, especially with regard to MVP vs. MVR (3). For non-infected mitral valvular diseases, such as myxomatous, ischemic or degenerative valve disease, MVP is prefered due to fewer reoperation, thromboembolism, and valve infection events (4). While for patients with IE, although several studies have confirmed a better survival after MVP compared with MVR in patients with IE, the small sample size and lacking of randomized controlled trials bring the bias and reduce the confidence level. Besides, so far only short and long-term survival were mentioned in published systematic review and the core issues of reinfection and reoperation were ignored (1, 5, 6). Therefore, in this paper, we performed this meta-analysis to evaluate whether MVP has better clinical outcomes than MVR for patients suffering from IE. Not only the survival but also core complications such as reinfection and reoperation were analyzed. To get more detailed information, the subgroup of patients with active IE was also analyzed.

## Methods

### Search strategy

Pubmed and Cochrane Library were searched for journals. “MVP”, “mitral valve plasty”, “mitral valve repair”, “mitral valve annuloplasty”, “mitral reconstruction”, “MVR”, “mitral valve replacement”, “infective endocarditis”, and “IE” were used either alone or in combination. The reference list of relevant articles and reviews were identified manually to find additional studies.

### Eligibility criteria

The inclusion criteria were as follows: (i) direct comparison of MVP vs. MVR; (ii) clinical outcomes (early survival, long-term survival, event-free survival, freedom from reoperation due to all-events, reinfection events) had to be provided in sufficient details to allow the extraction of hazard ratios(HR) or odd ratios(OR), and their standard errors or Kaplan–Meier curves. Two independent authors (Song Wang and Ting Zhou) extracted data from studies. Disagreements were resolved by a discussion with a superior (Dr. Yefan Jiang). Studies met the inclusion criteria were rated based on the NOS (7). Studies with a NOS score of 5 or lower were excluded. Paper quality was checked by Song Wang and Ting Zhou independently.

### Statistical analysis

Summary HR for event-free survival, freedom from reoperation due to all-events or reinfection, long-term survival, and OR for reinfection events and early mortality were obtained as weighted averages of measures from the induvidual studies, with inverse variances used as weights. We used methods provided by Parmer, Williamson and Tierney (8–10) to calculate the estimated HR and variance. Besides, we used a *Q*-statistic and *I*^2^ (index of inconsistency) test to quantify the heterogeneity degree and when significant heterogeneity (*P* < 0.1 or *I*^2^ > 50%) existed, a random effects model was applied. We omitted each study included in sequence to conduct the sensitivity analyses and visual inspection of funnel plots was used to assess the publication bias. RevMan 5.3 was used to analyze the data.

## Results

### Study search

**Figure 1:**
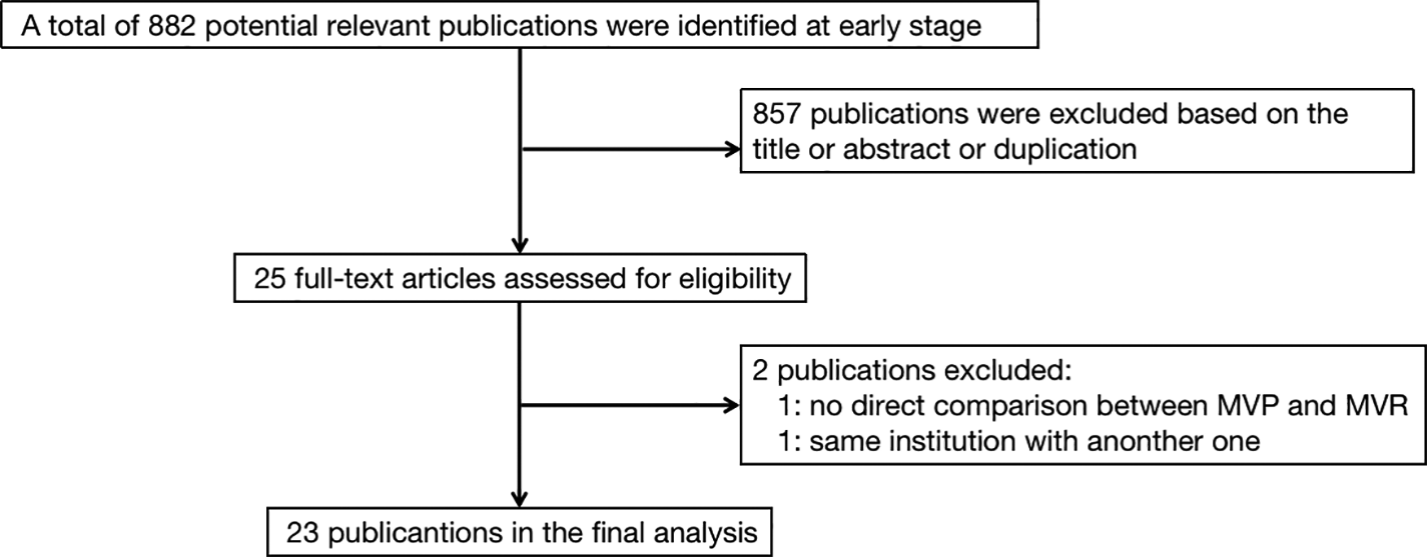
The flowchart outlining the literature search process.

The selection strategy in shown in Figure 1. 23 retrospective studies met the inclusion criteria. 25,615 patients were included in the final analysis, 10,719 of whom received MVP and 14,896 received MVR from 1980 to 2017. Table 1 presents the individual studies' characteristics.

**Table 1:** The characteristics of the individual studies.

Study	Country	Study period	Surgery							Active/ Healed	Mean age (years)		Male	
			MVP				MVR				MVP	MVR	MVP	MVR
Hyoung Woo chang 2014 (11)	South Korea	2004–2011	11				15			Both	38.2	48.3	9/2	11/4
Michele Musci 2010 (12)	Germany	1996–2007	61				166			Active	47.7	56.2	40/21	134/85
Markus J 2004 (13)	Switerland	1980–1996	57				97			Both	–	–	–	–
Tomislav Mihaljevic 2004 (14)	United States	1992–2002	21				32			Active	66	50	16	24
Leonid Sternik 2002 (15)	United States	1986–1999	16				28			Active	–	–	–	–
Thitipong Tepsiwan 2019 (16)	Tailand	2006–2017	38				76			Active	44.1	47.5	21	57
Eric shang 2009 (17)	United States	2002–2007	56				33			Both	48		59	
Derek D 1997 (18)	United States	1985–1995	Both	102					44	Both	51.9		100	
			Active	26					32					
			Healed	72					12					
Takashi Miura 2014 (19)	Japan	1999–2012	36				21			Active	57	55	35	27
Hsiu-An Lee 2018 (20)	Taiwan China	2005–2015	38				33			Active	42.3	53.7	24	20
Mohamad Alkhouli 2019 (21)	United States	2003–2016	7451				27204			Both	53	55	5000	16,513
Anton Tomsic 2017 (22)	Netherlands	2000–2016	51				32			Active	55	60	38	17
James S 2005 (23)	United States	1994–2003	Both		1882			4565		Both	56		3956	
			Active		423			2231			54		1547	
			Treated		1459			2111			58		2184	
Silvia Solari 2018 (24)	Belgium	1991–2015	155				37			Active	60.1	64.6	109	18
Sossio Perrota 2017 (25)	Sweden	2000–2015	76				64			Both	60	62	55	45
Rufin J. Defauw 2020 (26)	Netherlands	2000–2017	97				53			Active	57	61	20	22
Gregorio P. Cuerpo 2019 (27)	Spain	2008–2016	68				301			Active	–	–	–	–
Jose L. Navia 2019 (28)	United States	1988–2017	52				86			Both	55	58	44	52
Tom Kai Ming Wang 2014 (29)	New Zealand	2005–2011	25				35			Active	43.1	52.1	14	21
Hiroichiro Yamaguchi 2006 (30)	Japan	1999–2005	14				7			Both	58	53		
Sung-Ho Jung 2011 (31)	South korea	1994–2009	41				61			Active	34.4	43.1	19	33
Nana Toyoda 2017 (32)	United States	1998–2014	367				1603			Active	56.9	54.9	243	900
Elfriede Ruttmann 2005 (33)	Austria	1992–2004	34				34			Active	51.5	53.2	22	17

MVP, mitral valve plasty; MVR, mitral valve replacement.

### Early mortality

**Figure 2:**
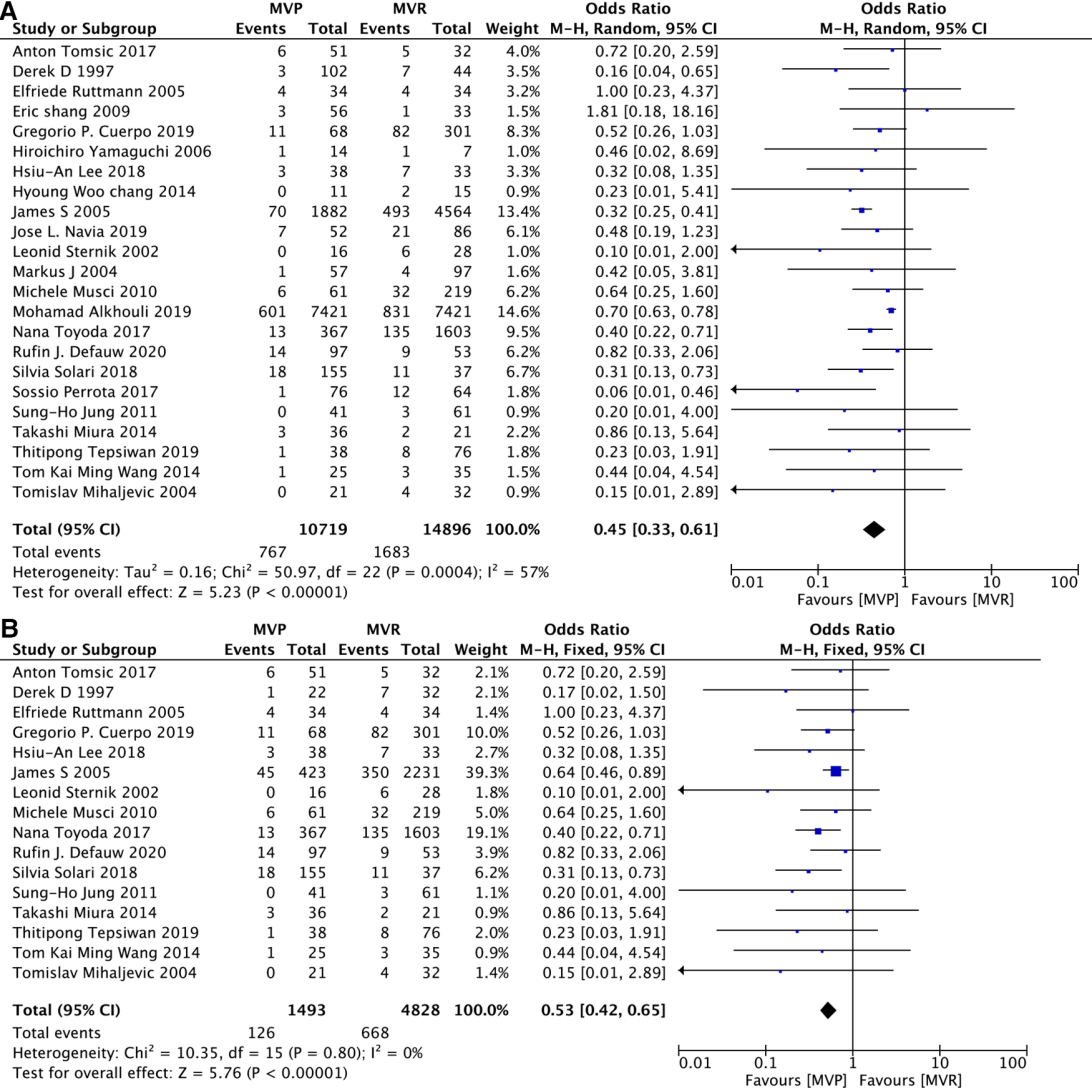
Meta-analysis for early mortality: (**A**) MVP vs. MVR; (**B**) MVP vs. MVR (active IE); MVP, mitral valve plasty; MVR, mitral valve replacement.

Early death refers to in-hosptial death or death occurring within 30 days after operation. All 23 studies contained related details on it and a random effects model was used because of the relatively high heterogeneity (*I*^2^ = 57%, *P* = 0.0004) among studies included. Lower early mortality was apparent in MVP group. Among those 23 studies, 16 studies (12, 14–16, 18–20, 22–24, 26, 27, 29, 31–33) reported patients with active IE. The advantages on early mortality of MVP over MVR was also obvious, when only patients with active IE were included. [MVP vs. MVR: OR: 0.45, 95% CI: 0.33–0.61, Figure 2A; MVP vs. MVR (active IE): OR: 0.53, 95% CI: 0.42–0.65, Figure 2B].

Among those 23 studies, 3 studies contain 90% percentage of patients (21, 23, 32), the exclusion of any one or two or all of those three studies didn’t change the overwhelming of MVP. (As shown in Supplementary Figure S1).

### Long-term survival

**Figure 3:**
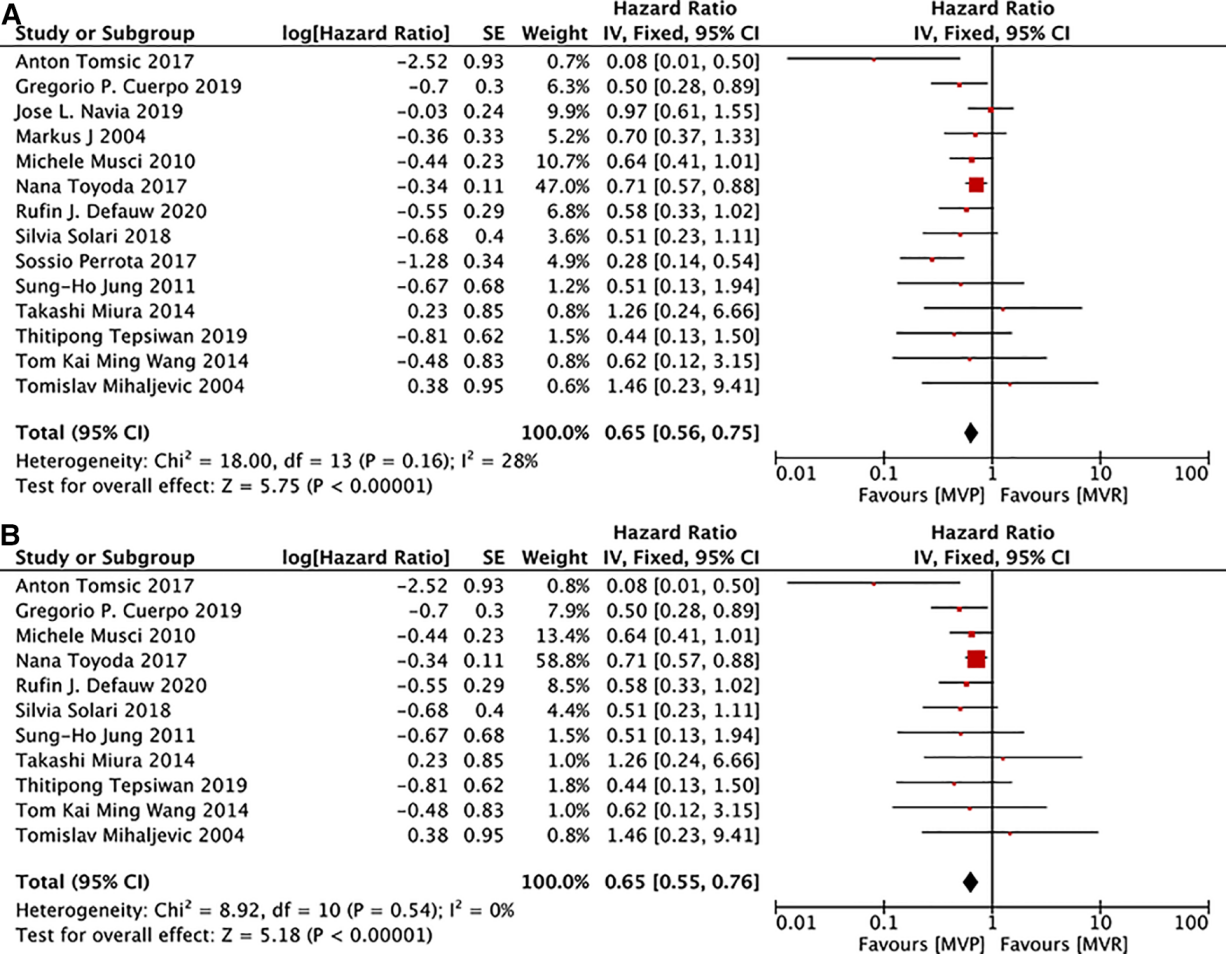
Meta-analysis for long-term survival: (**A**) MVP vs. MVR; (**B**) MVP vs. MVR (active IE); MVP, mitral valve plasty; MVR, mitral valve replacement.

15 studies (12–14, 16, 19, 22, 24–29, 31, 32) provided related data. Although there was significant heterogeneity(*I*^2^ = 47%, *P* = 0.02) among those studies, heterogeneity can be accepted after omiting the study of Eric Shang et al*.* (17), and deletion of that study did not change the overall results. 11 studies (12, 14, 16, 19, 22, 24, 26, 27, 29, 31, 32) documented details of patients with active IE. The results presented that patients receiving MVP were with a decreased long-term risk of death irrespective of whether only patients with active IE were included. [MVP vs. MVR: HR: 0.61, 95% CI: 0.49–0.75, Figure 3A; MVP vs. MVR (active IE): HR: 0.65, 95% CI: 0.55–0.76, Figure 3B].

### Event-free survival

**Figure 4:**
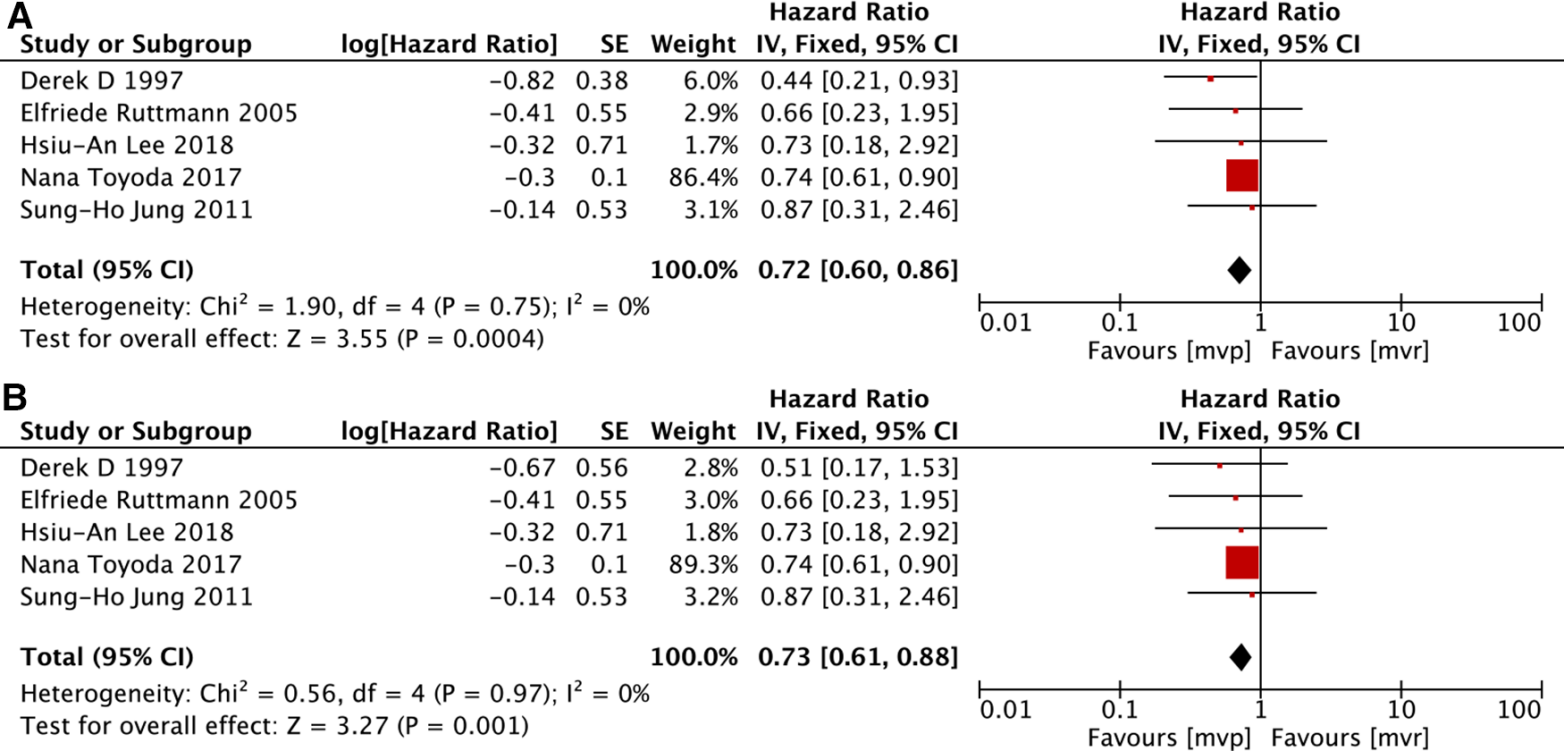
Meta-analysis for event-free survival: (**A**) MVP vs. MVR; (**B**) MVP vs. MVR (active IE); MVP, mitral valve plasty; MVR, mitral valve replacement.

Here event-free means freedom from cerebravascular disease, valvular reoperation, recurrence of endocarditis, and death (33). Of the 23 included studies, 6 (12, 18, 20, 31–33) provided information to allow the determination of event-free survival but significant heterogeneity existed (*I*^2^ = 48%, *P* = 0.09), much smaller heterogeneity could be calculated after removing the study of Michele Musci et al*.* (12), and the deletion of that study did not change the overall results. All of the 6 studies also provided details related to patients with active IE. Same as the analysis mentioned above, significant heterogeneity existed and the deletion of the study by Michele Musci et al*.* (12), could reduce the heterogeneity and didn't change the overall results. The analyses demonstrated higher event-free survival in the MVP group for both the whole patients and active IE subgroup. [MVP vs. MVR: HR: 0.72, 95% CI: 0.60–0.86, Figure 4A; MVP vs. MVR(active IE): HR: 0.73, 95% CI: 0.61–0.88, Figure 4B].

### Freedom from reoperation due to all-events

**Figure 5:**
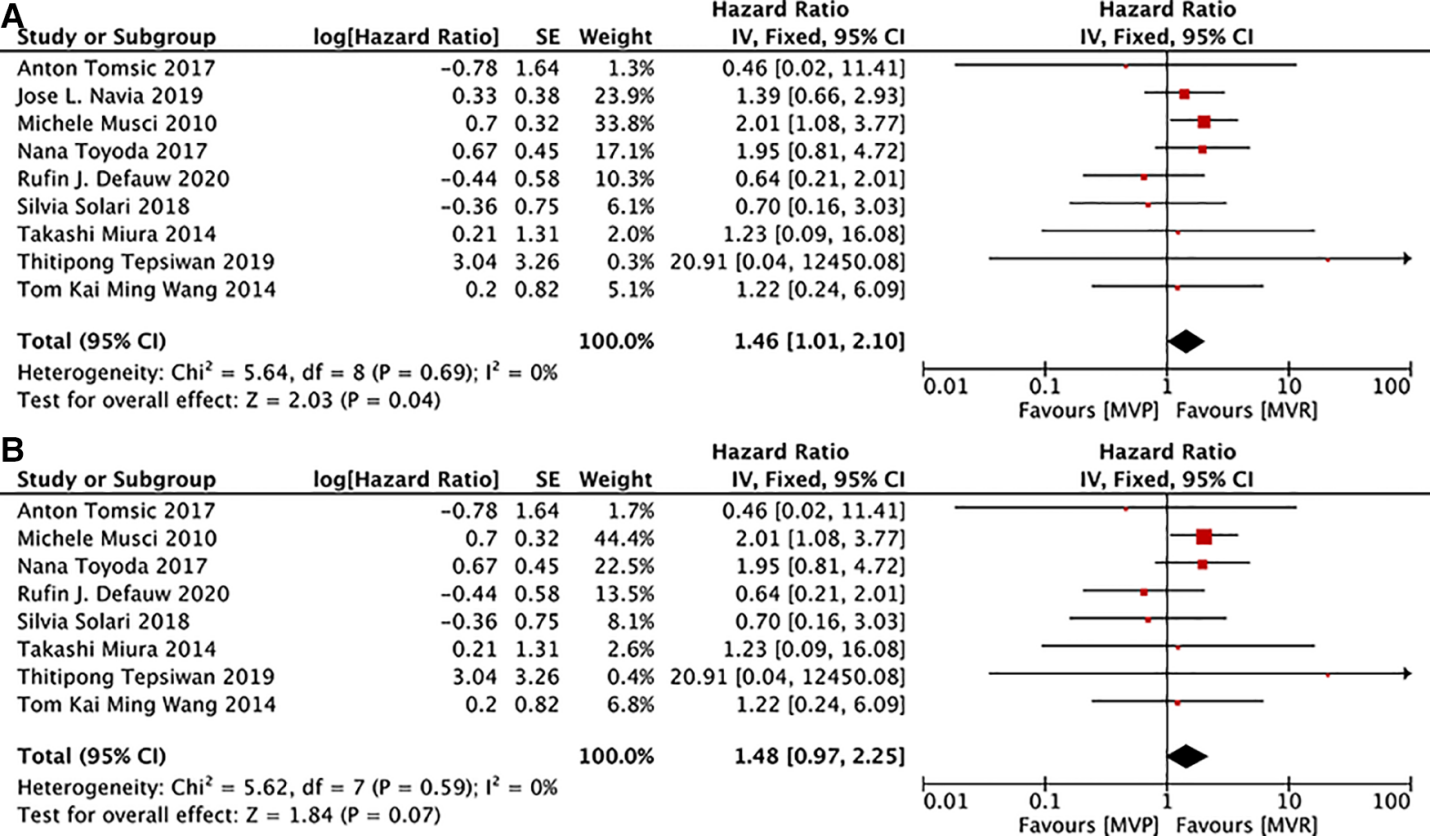
Meta-analysis for freedom from reoperation due to all-events: (**A**) MVP vs. MVR; (**B**) MVP vs. MVR (active IE); MVP, mitral valve plasty; MVR, mitral valve replacement.

9 studies (12, 16, 19, 22, 24, 26, 28, 29, 32) presented details on the analysis of freedom from reoperation due to all-events, 8 (12, 16, 19, 22, 24, 26, 29, 32) of which documented information related to active IE. While the summary HR suggested that reoperation rate due to all events was lower in patients with IE following MVR, no significant differences existed in patients with active IE after MVR and MVP. [MVP vs. MVR: HR: 1.46, 95% CI: 1.01–2.10, Figure 5A; MVP vs. MVR (active IE): HR: 1.48, 95% CI: 0.97–2.25, Figure 5B].

### Reinfection events

**Figure 6:**
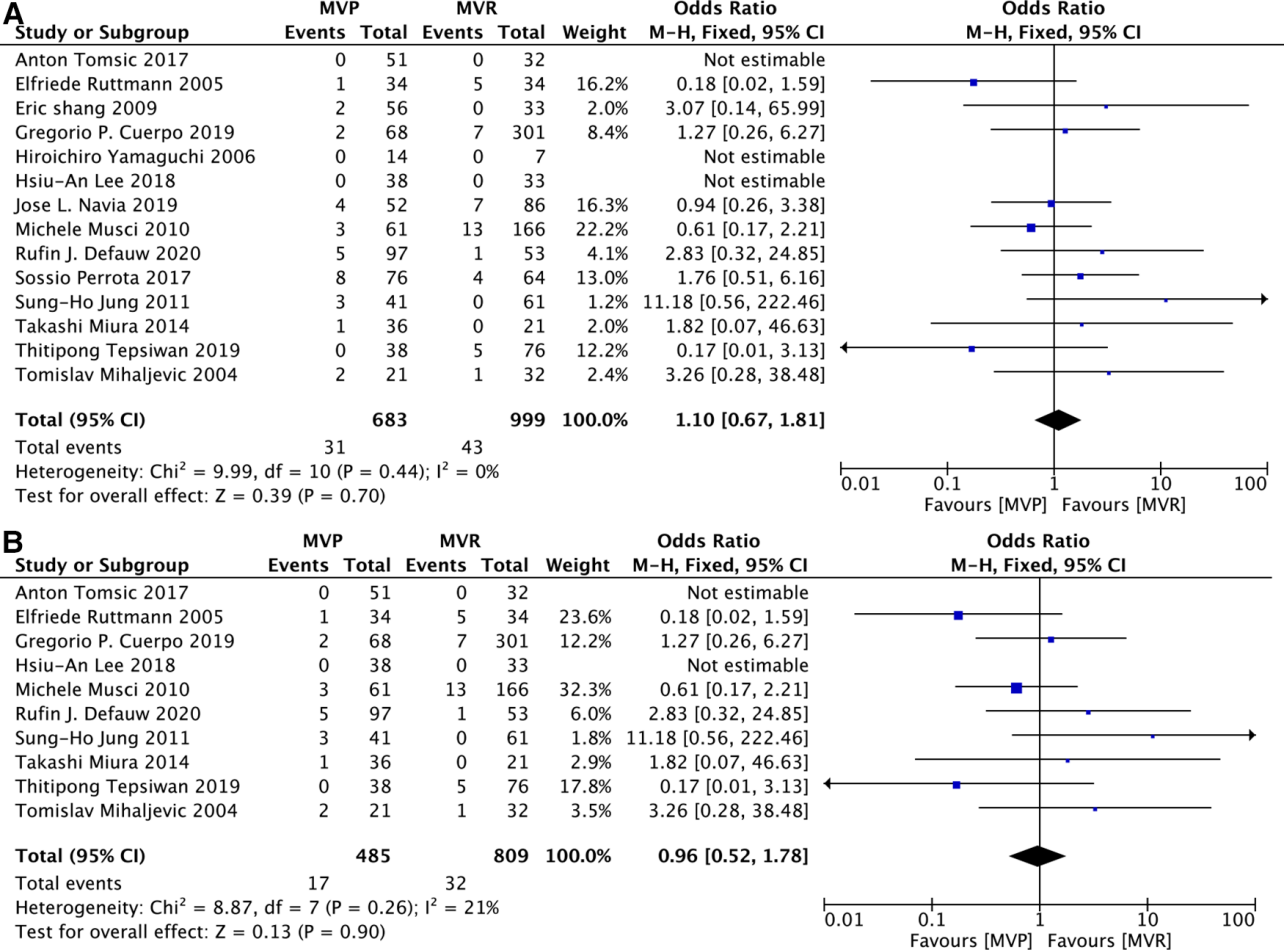
Meta-analysis for reinfection events between MVP and MVR; (**A**) MVP vs. MVR; (**B**) MVP vs. MVR (active IE); MVP, mitral valve plasty; MVR, mitral valve replacement.

Information on reinfection events was obtained from 14 studies (12, 14, 16, 17, 19, 20, 22, 25–28, 30, 31, 33). 10 (12–14, 16, 19, 20, 26, 27, 31, 33) of which provided details related to patients with active IE. No differences in reinfection events between those two groups were observed no matter whether only active IE patients were included. [MVP vs. MVR: OR: 1.10, 95% CI: 0.67–1.81, Figure 6A; MVP vs. MVR (active IE): OR: 0.96, 95% CI: 0.52–1.78, Figure 6B].

And there is still no difference between in reinfection between MVP group and MVR group when we take time into account and calculate the HR value of reinfection. 4 studies (26, 28, 29, 32) provided related details and 3 (26, 29, 32) of which provided details related to patients with active IE. [MVP vs. MVR: HR: 1.91, 95% CI: 0.53–6.87, Supplementary Figure S2A; MVP vs. MVR (active IE): HR: 1.39, 95% CI: 0.18–10.63, Supplementary Figure S2B].

## Discussion

The incidence of IE is gradually increasing (34). IE patients with symptoms of severe valve dysfunction, heart failure, recurrent systemicembolization, et al. should receive surgical treatmen (35). Although the diagnosis, medical treatment and surgical techniques have improved, mortality and morbidity still remain high (2).

Repair and replacement are both candidate therapeutic methods for valve diseases. More and more surgeons prefer MVP as the first choice for patients with myxomatous, ischemic or degenerative mitral valve diseases, due to MVP's advantages of lower early morbidity and operative mortality, higher long-term survival, and fewer reoperation events (4). However, the superiority of MVP in patients with IE still needs identification furtherly. So far related papers are still limited. In published papers of this topic, several limitations do exist. Firstly, there were only retrospective studies. Secondly, only small number of cases were included in some single-center studies (36). Thirdly, in multi-center studies, patients were included through clinical codes and selection bias was large. Besides, the gap of medical level between different hospitals reduced the reliability of the final results and the rate of successful MVP in active IE varied significantly among previous literatures from 15% up to 100% (16).

MVP is much more complicated in the presence of IE accompanied with the valves' primary pathology like degeneration, calcification, et al. (36). Concomitant cardiac abnormalities such as aortic valve IE, coronary artery diseases, et al. also contribute (36, 37). For patients with active IE, the reconstructive surgery in inflammatory tissue may be difficult and recurrence of infection is relatively common (5). For patients with healed IE, MVP may not be feasible because of the valvular destruction and heart failure resulting from the infectious process (1). Therefore, two concerns arise on the surgical strategy choice of patients with IE: reinfection for patients with active IE and reoperation for patients with healed IE (26). Previous systematic reviews focused less on those two concerns and had no distinguishing between clinical outcomes of patients with active IE and healed IE (1, 5). Only two published papers provided details related to MVP and MVR in healed IE subgroups. Therefore we can only infer the relevant conclusions from comparison of clinical outcomes between all patients and subgroup of active IE patients following different mitral strategies.

Analysis showed that early survival, late survival and event-free survival were higher in the MVP group no matter whether only patients with active IE were included. It does make sense. Firstly, MVP can provide preservation of the subvalvular apparatus and protection of the left ventricular function, which can avoid death resulting from impaired left ventricular function. Secondly, MVP usually accompanies with much more physiologic fitting hemodynamics which can make contributions to the recovery of left ventricular function. Thirdly, the possiblity of valve-related events and perivalvular leakage was reduced by the existence of autogenous valves. Fourthly, it helps patients avoding thromboembolism and hemorrhage with no need for long-term use of anticoagulant therapy (4). Besides, surgeons' preference for MVP in patients with superior body condition and less damaged valves also contribute to fewer complications and death, which can be reflected by the fact that patients receiving MVP are normally younger.

No difference exists in reinfection between MVP group and MVR group no matter whether patients with active IE were regarded as a subgroup. Traditionally, completion of a full course of antibiotics before surgery was recommended (38). While for patients with refractory congestive heart failure, uncontrolled sepsis, et al., mindless delay of surgery should be avoided (39). Moreover, recent literatures have suggested that early intervention may bring benefits like fewer cardiovascular events and less damaged valvular structure (17, 40). Actually more and more patients are receiving surgeries during active phase. MVR has been the standard procedure for patients with acute IE due to the demand of complete excision of infective tissue (18). However, we can't neglect the fact that the grafts themselves are susceptible to becoming the source of infection (6). There is still debate on the implantation of artificial material in active IE (13, 33). Two adverse events may occur. One is bacterial colonization prior to endothelization of prosthetic materials, and the other is late prosthetic valve IE (22). Some scholars even suggested that, when possible, all artifical materials should be avoided to reduce the recurrence of IE (41, 42). However, there is still no difference between in reinfection between MVP group and MVR group when we take time into account and calculate the HR value of reinfection. Sung-Ho Jun and Moon MR indicated that the complete excision of infective material is much more important than avoiding prosthetic material in preventing recurrence (31, 43). Anyway, we should not ignore either the role of residual infected tissue or artificial implants in reinfection. A successful MVP for patients of IE should comprise of thorough resecting of infected tissue and minimal artificial material implantation at the same time, which requires experience in repair.

Although surgery is important, pre- and post-operative antibiotic treatment is also essential for IE therapy, especially for those patients of active phase. It is necessary for patients to receive effective antimicrobial treatment which may decrease the positive cultures from explanted valves after surgery (44). Increasingly invasive health care intervention has changed the distribution of bacteremia in IE patients which makes culture result more important (45). Song Wan reported that 85% of IE cases were culture-positive before operations. And operative specimens are particularly valuable for those cultutre-negative ones (35, 36). 6-weeks duration of intravenous antimicrobial treatmet is recommended following surgical intervention and longer treatment is necessary when invasive infections, difficult-to-treat microorganisms and prosthetic materials infections exist (35).

While the reoperation rate due to all events was lower in patients with IE following MVR, no significant differences exist in patients with active IE following mitral intervention. It can be concluded that the reoperation rate due to all events was lower in patients with healed IE following valve replacement rather than repair. And mitral insufficiency accounts for most of those reoperations (46). For patients with active IE, similar reoperation rate is due to less damaged valvular structure and better physical and cardiac condition in early infective stage (39). While for patients with healed IE, more reoperation events in MVP group may be the result of the more extensive destruction of infective valve tissue, annulus and/or the subannular apparatus (47). Wan and associators reported that while vegetation was the most common pathogen in acute IE, valve prolapse resulted from chordae rupture was the most common one in healed IE (36). Repairs in the presence of healed but destructive valves are inevitably more complicated.

Here we have to mention annular abscess as a special pathogen. It represents much more serious situation, but fortunately, it is uncommon. Wan reported that annular or paravalvular abscess was reported in less than 10% of patients (36). Due to the complexity of the anatomy, radical debridement, sterilization and drainage of the infected area are more difficult. Plasty needs more experience and skills, and patches must be generous to minimize tension on the suture lines. Replacement without additional reconstruction is also a good choice. The valve prosthesis could be anchored to the ventricular muscle or to the reconstruction patch in a way that prevents leakage and pseudoaneurysm development beneath the prosthesis. And of course, annular reconstruction is still needed when it is necessary during replacement procedures (48).

The MVP approach is patient-specific (11). Surgeons performs valvular repairs for IE patients mostly based on their experience of noninfectious valve disease repair (36). No guidelines have been raised and repairs in infectious valves are much more complicated. The ratio and underlying patients of MVP varied greatly among different surgical teams and centres. While some centres can perform difficult repair, including annular abscesses (33), some other centres only perform MVP in patients with vegetations alone (14). This does increase the heterogeneity of our research. The surgeon's experience in MVP is crucial. Only surgeons with rich experience and sufficient skills are capable of repairing severely destructed mitral valves (33). At the same time, strict patient selection is also essential. It's important to distinguish patients that are likely to be repaired from the patients that may have poor repair results.

### Limitations

Several limitations exist in this analysis. Firstly, only retrospective studies were included in this study. Secondly, the operative years were of a broader range which might have implications in terms of the comparability of studies included in this analysis. Thirdly, there were only a few patients in some analyses which may resulted in higher selection bias. Fourthly, the mechods and technics of MVP were varied in all studies, mainly depanding on surgeons’ experience. Fifthly, no direct comparison between MVP and MVR in patients with healed IE.

## Conclusions

To sum up, MVP and MVR are both beneficial for patients suffering from IE. Both two therapeutic methods have their own advantages and disadvantages. It is still hard to determine which one is better. For surgeons with consummate skills, MVP can be the preferred choice for suitable patients suffering from IE. And active phase is not a disadvantageous factor in MVP. More randomized controlled trials should be conducted.

## Data Availability

The raw data supporting the conclusions of this article will be made available by the authors, without undue reservation.
