# Corrigendum: Clinical outcomes following surgical mitral valve plasty or replacement in patients with infectious endocarditis: A meta-analysis

**DOI:** 10.3389/fsurg.2023.1142744

**Published:** 2023-02-07

**Authors:** Song Wang, Ting Zhou, Jinhui Bian, Geng Li, Wenjing Zhang, Si Chen, Yefan Jiang

**Affiliations:** ^1^ Department of Cardiovascular Surgery, The Affiliated Taizhou People’s Hospital of Nanjing Medical University, Taizhou School of Clinical Medicine, Nanjing Medical University, Nanjing, China; ^2^ Department of Cardiovascular Surgery, The First Affiliated Hospital of Nanjing Medical University, Nanjing, China; ^3^ Health Management Center, The Central Hospital of Wuhan, Tongji Medical College, Huazhong University of Science and Technology, Wuhan, China; ^4^ Department of Cardiovascular Surgery and Heart Transplantation, Union Hospital, Tongji Medical College, Huazhong University of Science and Technology, Wuhan, China; ^5^ Department of Ultrasound Medicine, The Second Afliated Hospital of Harbin Medical University, Harbin, China; ^6^ Department of Ultrasound Medicine, Union Hospital, Tongji Medical College, Huazhong University of Science and Technology, Wuhan, China

**Keywords:** infectious endocarditis, mitral valve plasty, mitral valve replacement, clinical outcomes, meta-analysis

Corrigendum on: Clinical outcomes following surgical mitral valve plasty or replacement in patients with infectious endocarditis: A meta-analysis By Wang S, Zhou T, Bian J, Li G, Zhang W, Chen S and Jiang Y. (2023) Front. Surg. 9:1048036. doi: 10.3389/fsurg.2022.1048036

**Incorrect Affiliation**

In the published article, there was an error in affiliation [1]. Instead of “The Affiliated Taizhou People's Hospital of Nanjing Medical University”, it should be “Department of Cardiovascular Surgery, The Affiliated Taizhou People's Hospital of Nanjing Medical University, Taizhou School of Clinical Medicine, Nanjing Medical University, Nanjing, China”.

The authors apologize for this error and state that this does not change the scientific conclusions of the article in any way. The original article has been updated.

**Text Correction**

In the published article, there was an error. We forgot to state the symbol † means those three authors contributed equally to this work. The corrected sentence appears after the author affiliation and before background and reads:

“† those three authors contributed equally to this work”

The authors apologize for this error and state that this does not change the scientific conclusions of the article in any way. The original article has been updated.

**Text Correction**

In the published article, we excluded some key words. The sentence previously stated:

“infectious endocarditis, mitral valve repair, mitral valve replacement, clinical outcomes, meta-analysis infectious endocarditis, meta-analysis”

The corrected sentence appears below:

“infectious endocarditis, mitral valve plasty, mitral valve replacement, clinical outcomes, meta-analysis”

The authors apologize for this error and state that this does not change the scientific conclusions of the article in any way. The original article has been updated.

