# Supplementary material for: Clinical outcomes following surgical mitral valve plasty or replacement in patients with infectious endocarditis: A meta-analysis
Source: Front Surg. 2023 Jan 6;9:1048036. doi: 10.3389/fsurg.2022.1048036 (PMC9869952; doi:10.3389/fsurg.2022.1048036)
Supplement: Supplementary file 1 [file Datasheet1.docx]

Supplementary Material

# Supplementary Figures and Tables

For more information on Supplementary Material and for details on the different file types accepted, please see [here](http://home.frontiersin.org/about/author-guidelines#SupplementaryMaterial). Figures, tables, and images will be published under a Creative Commons CC-BY licence and permission must be obtained for use of copyrighted material from other sources (including re-published/adapted/modified/partial figures and images from the internet). It is the responsibility of the authors to acquire the licenses, to follow any citation instructions requested by third-party rights holders, and cover any supplementary charges.

## Supplementary Figures


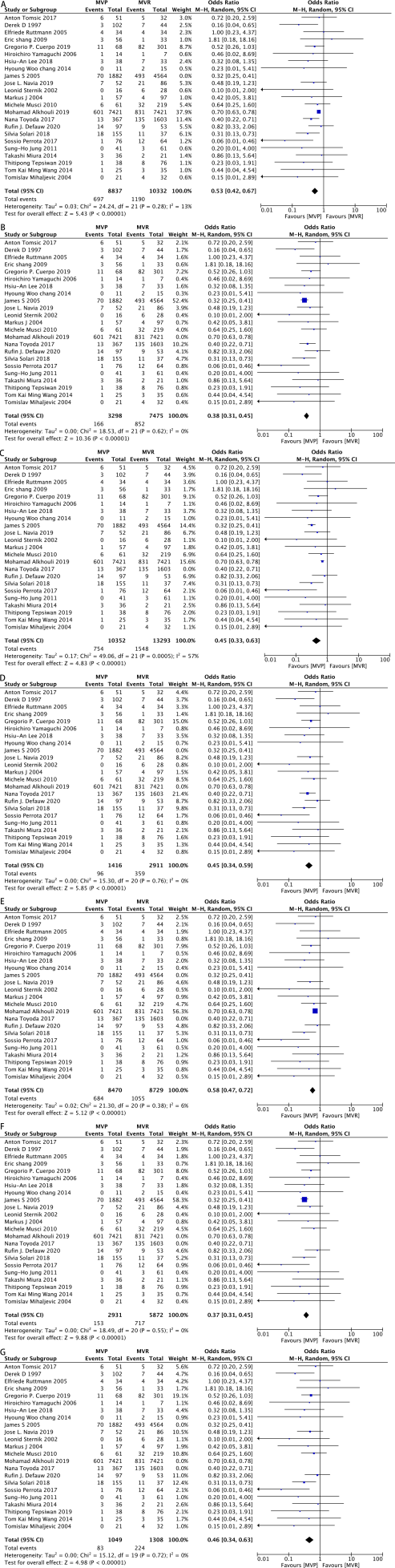


**Supplementary Figure 1**

Meta-analysis for early mortality after the exclusion of any one or two or all of studies by Mohamad Alkhouli *et al,* James S *et al* and Nana Toyoda *et al.*

A: MVP vs MVR(the exclusion of the study by James S *et al*);

B: MVP vs MVR(the exclusion of the study by Mohamad Alkhouli *et al*);

C: MVP vs MVR(the exclusion of the study by Nana Toyoda *et al*);

D: MVP vs MVR(the exclusion of the studies by James S *et al* and Mohamad Alkhouli *et al*);

E: MVP vs MVR(the exclusion of the studies by James S *et al* and Nana Toyoda *et al*);

F: MVP vs MVR(the exclusion of the studies by Mohamad Alkhouli *et al* and Nana Toyoda *et al*);

G: MVP vs MVR(the exclusion of the studies by James S *et al,* Mohamad Alkhouli *et al* and Nana Toyoda *et al*);

MVP: mitral valve repair, MVR: mitral valve replacement


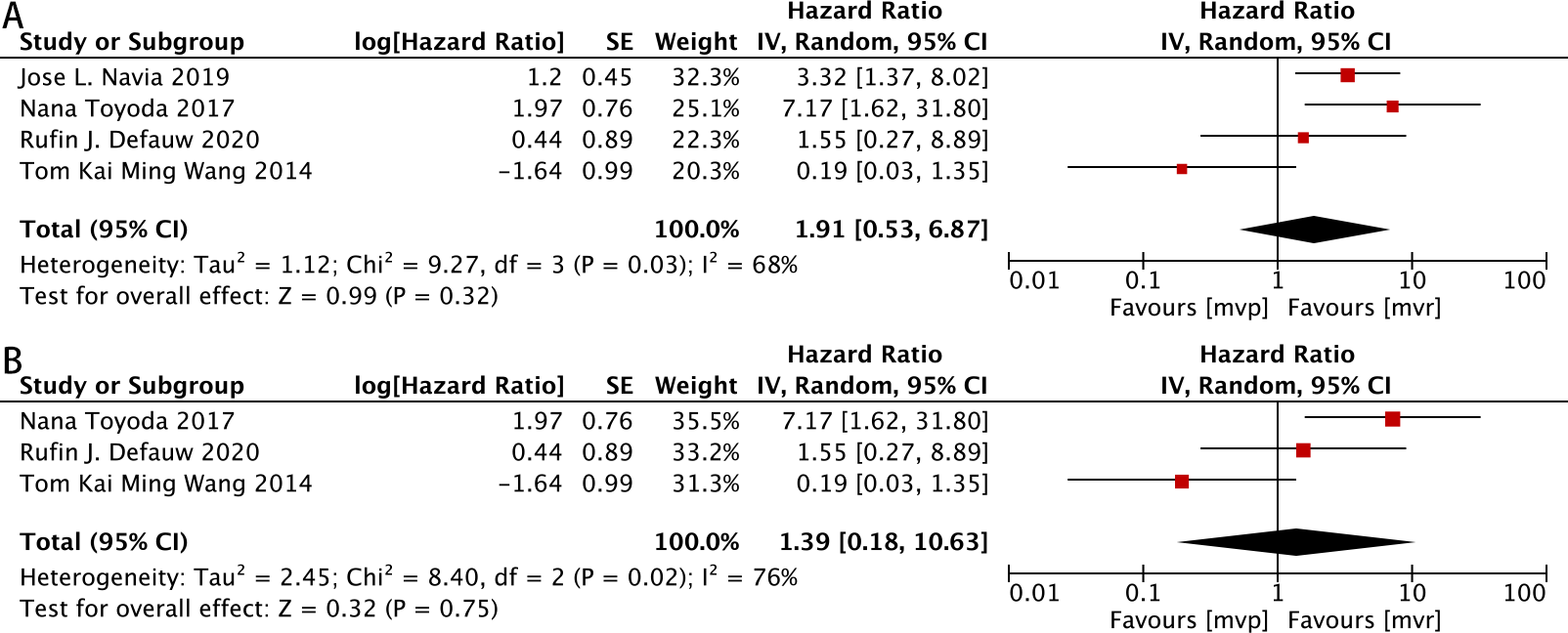


**Supplementary Figure 2**

Meta-analysis for reinfection events between MVP and MVR; A: MVP vs MVR; B: MVP vs MVR(active IE); MVP: mitral valve repair, MVR: mitral valve replacement
